# Technical challenges of studying early human development

**DOI:** 10.1242/dev.201797

**Published:** 2023-06-01

**Authors:** Peter J. Rugg-Gunn, Naomi Moris, Patrick P. L. Tam

**Affiliations:** ^1^ Epigenetics Programme, Babraham Institute, Cambridge CB22 3AT, UK; ^2^ Centre for Trophoblast Research, University of Cambridge, Cambridge CB2 3EG, UK; ^3^ Wellcome-MRC Cambridge Stem Cell Institute, Cambridge CB2 0AW, UK; ^4^ The Francis Crick Institute, London NW1 1AT, UK; ^5^ Embryology Research Unit, Children's Medical Research Institute, The University of Sydney, Westmead NSW 2145, Australia; ^6^ School of Medical Sciences, Faculty of Medicine and Health, The University of Sydney, Sydney NSW 2006, Australia

**Keywords:** Embryo, Stem cell models, *In vitro* development, Fidelity, Reproducibility

## Abstract

Recent years have seen exciting progress across human embryo research, including new methods for culturing embryos, transcriptional profiling of embryogenesis and gastrulation, mapping lineage trajectories, and experimenting on stem cell-based embryo models. These advances are beginning to define the dynamical principles of development across stages, tissues and organs, enabling a better understanding of human development before birth in health and disease, and potentially leading to improved treatments for infertility and developmental disorders. However, there are still significant roadblocks *en route* to this goal. Here, we highlight technical challenges to studying early human development and propose ways and means to overcome some of these constraints.

## Teaser

**Summary:** Identifying technical challenges of studying human development, and potential solutions to those obstacles, provides a framework for the research community to help advance knowledge and application of human developmental biology.

## Introduction

Early human development is defined as the first 8 weeks after conception: a period that covers major landmarks in embryogenesis towards the establishment of a healthy pregnancy. Weeks 1 to 3 incorporate embryo implantation, specification of the germ layers and the germ line, and establishment of the early body plan. Weeks 3 to 5 represent early organogenesis stages, where progenitors and their derived cell types in the major organ systems are formed, which is accompanied by morphogenetic patterning, such as the closure of the neural tube and looping of the heart. From weeks 5 to 8, organogenesis continues to establish more-mature organs, such as skeletogenesis in the limb buds and formation of the spine, alongside overall body growth. After this point, the embryo, which has acquired species-specific anatomy and functional attributes, is considered a fetus for the rest of its *in utero* development.

An informed understanding of the development of the embryo at this formative phase is important because the patterning events and cellular interactions that occur during this period are crucial for the structural organisation of tissues and organ systems that ultimately lead to viable progeny. Knowledge of normal development is also informative of the potentially catastrophic effects of perturbations of development, which can lead to pregnancy loss and congenital malformations.

**Fig. 1.**
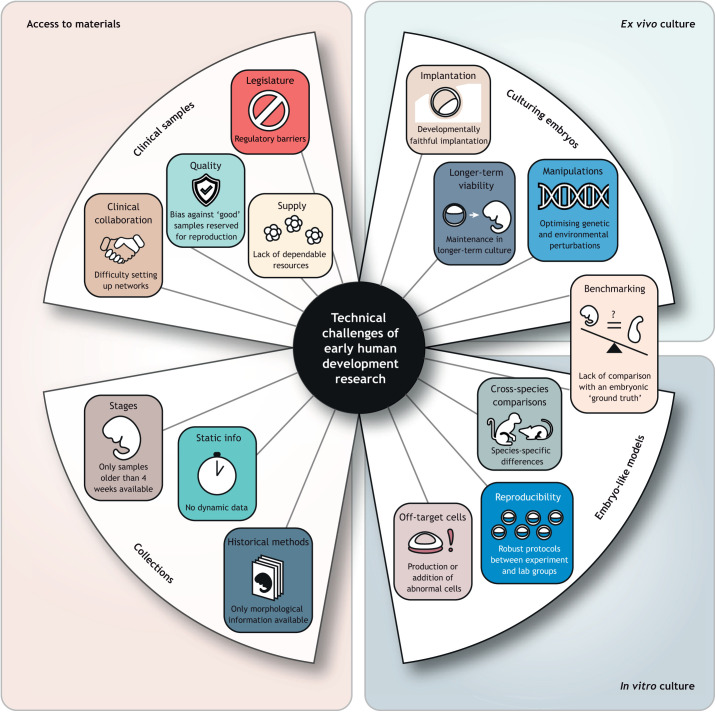
**Major technical challenges of studying early human development.** Some of the challenges facing researchers include the broad categories of access to research materials, which includes both accessing clinical samples directly (upper left) and extant collections of embryonic and fetal material, such as the Kyoto and Carnegie collections (lower left), as well as challenges of *ex vivo* culture of pre-implantation embryos (upper right) and *in vitro* culture of embryo-like models (lower right). Each of these categories faces distinct technical challenges, but many also present opportunities to improve with technical advances, scientific progress, concerted community efforts and improved regulatory frameworks.

Towards these goals, recent technological breakthroughs have enabled discovery research that was not previously feasible, such as measuring and modelling tissue patterning and morphogenetic events in increasingly rich cellular and molecular detail. And yet significant challenges remain and will need to be addressed in order to glean a deeper insight into this window in development. Although this Spotlight focuses only on technical aspects (Fig. 1), these are inextricably linked to the ethical and legal challenges of human developmental research, and we direct the reader towards several excellent reviews on these topics (Ismaili M'hamdi et al., 2022; Matthews et al., 2021; Pereira Daoud et al., 2020).

## Access to embryonic materials

The best way to understand the development of human embryos would, of course, be to study the human embryo itself. This research is enabled by generous donation of material specifically for the purposes of research, which permits researchers to perform high-quality characterisation of human embryos across different developmental stages. However, this valuable opportunity can also represent the biggest technical hurdle in studying early human development. There is still a barrier to accessing of human embryonic and fetal material, and, despite the frequent willingness of individuals to donate surplus embryos to research (Samorinha et al., 2014), clinical-research frameworks are often lacking, which then limits opportunities for embryo research. For example, although it is possible to obtain preimplantation embryos through the donation of embryonic material generated by *in vitro* fertilization (IVF), significant regulatory considerations, including differences across jurisdictions, can create high entry barriers for research (Lovell-Badge et al., 2021). Furthermore, the reliance on donated IVF embryos raises issues of embryo quality, as embryos of assumed high quality (based mainly on morphological measurements) are saved for reproductive purposes, leaving those of lesser quality, deemed not suitable for reproduction purposes, for donation to research (Dennis et al., 2006). There is also a need to access a sustainable source of experimental materials that is crucial for long-term continuing research, so supporting the pathways that enable embryo donation are vital. Setting up and maintaining the material-transfer processes requires significant investment by the research teams and their collaborating fertility clinics. This can also lead to the underuse, and in some cases loss, of embryonic materials for research. As potential solutions, more efficient processes could be imagined, such as the establishment of strategic, collaborative interactions and simplified processes to allow sharing of donated materials between laboratories.

Biobanks for archiving fetal tissues from pregnancy terminations, typically available between 4 and 20 weeks post-conception, provide a resource of experimental materials underpinning scientific investigation of embryos at postimplantation stages. An example is the Human Developmental Biology Resource, which collects and stores embryonic and fetal tissues through appropriate ethical and legal review processes, and supplies samples to registered research projects. This is an efficient way to oversee and distribute precious samples that are in limited supply. Support for biobanks requires stable long-term funding and strong scientific advocacy, both for existing biobanks and for prioritising new biobanks to meet specific demand. Centralised banking of week 1-8 embryonic materials donated for research may overcome the obstacle of sourcing and coordinating the supply of embryonic materials for research on early human development.

Likewise, studies of historical human embryo collections have provided invaluable information about the anatomical characteristics of developing embryos, and yet there are several major technical limitations of these resources. For example, the collections have provided structural image data of the embryo either *in toto* or from histological sections, which primarily capture gross morphological features of the samples. The datasets of these archival materials are also inherently static. Encouragingly, recent studies have shown that some archived samples from biobanks are amenable to spatial and multiomic analysis (Arutyunyan et al., 2023; Zhang et al., 2023), indicating that the existing collections might be used to generate new insights by applying modern technologies that were not previously feasible.

Access to human materials between the peri-implantation and immediate postimplantation stages, between week 2 to week 4 of development, is significantly limited because it is often too early in pregnancy to acquire abortus material and the culture of human embryos beyond day 14 is forbidden in most jurisdictions (Mathews and Morali, 2020). Although one 14-17 day [Carnegie stage (CS)7] gastrula-stage embryo recently became available for embryological study (Tyser et al., 2021) and, likewise, a study reported the expression profiling of seven embryos spanning 4-6 weeks (CS12-CS16) (Xu et al., 2023), the scarcity of embryonic specimens at weeks 2-4 leaves a gap of knowledge of early development, precisely at the stage when the embryo is establishing the early body plan. Obtaining multiple embryos under 8 weeks with consistent quality and genome integrity is particularly impractical, making the study of this period of development especially challenging.

## Current experimental challenges for research directly on embryonic materials

When early embryonic materials are obtained, researchers are confronted with further technical challenges, including limitations associated with growing embryos *ex vivo* and in experimental tractability. In particular, identifying conditions to model the implantation of human embryos *in vitro* have been difficult to achieve. Although protocols exist for culturing human embryos over the second week of development (Deglincerti et al., 2016; Shahbazi et al., 2016; Xiang et al., 2020), these embryos lack the appropriate recapitulation of morphogenesis of embryos implanted *in vivo* and the absence of maternal tissues from the extra-embryonic structures raise concerns that the physiological equivalence of these post-blastocyst embryos might be compromised. To improve embryo implantation and development in culture, recent studies have co-cultured human blastocysts and stem cell-based blastoids with endometrial cells, which seem to facilitate attachment and could help to install the signals between trophectoderm and endometrium (Kagawa et al., 2022; Rawlings et al., 2021; Yu et al., 2022 preprint). Identifying conditions to culture postimplantation embryos that replicate the morphology of those *in vivo* is, therefore, a major goal. This challenge is further impeded by the current inability to compare *in vitro* embryo models directly with matching *in vivo* postimplantation embryos at the molecular level. For now, postimplantation human embryo cultures can be benchmarked against non-human primate embryos as a proxy (Bergmann et al., 2022; Cui et al., 2022; Nakamura et al., 2016, 2017; Zhai et al., 2022), but evident species-specific differences may confound such comparisons, and examples of this include differing transcriptional profiles during early development (Boroviak et al., 2018; Cui et al., 2022) and differences in the implantation of embryos into the endometrium (Siriwardena and Boroviak, 2022).

The difficulty in maintaining later stages (weeks 5-8) of embryo development *in vitro* for experimental embryology experiments also raises a technical challenge that limits the options for dynamic readouts, interventional experiments and lineage analysis. Although recent advances have enabled mouse embryos to be cultured for prolonged periods (Amadei et al., 2022; Tarazi et al., 2022), this has not yet been achieved for human embryonic samples. Current efforts to culture embryonic tissue explants are ongoing and can sustain tissues for a short period *in vitro*.

Finally, there are challenges regarding the ability to perform targeted genetic manipulation of human embryonic material, which are crucial methods for functional genomics studies and, for example, to generate cell-type and function reporters as means of experimental readout. Furthermore, the regulations in many jurisdictions that prohibit the genetic modification of human embryos for research purposes present an obstacle that impacts the scope of experimentation. A small number of studies have generated gene knockouts in human embryos (Fogarty et al., 2017; Stamatiadis et al., 2021, 2022), but these methods are technically challenging (e.g. often requiring significant protocol optimisation at each stage of the genetic modification process and hampered by the limited information about DNA repair processes in human embryos), and can lead to unintentional off-target genetic events (Alanis-Lobato et al., 2021; Zuccaro et al., 2020). In addition, such optimisation experiments that are necessary to improve the efficiency of genetic manipulations require the use of large numbers of embryos, which is prohibitive for many research groups. Where feasible, further investigation of DNA replication and repair pathways in early human development, combined with new CRISPR-based technologies, could be the solution to increasing editing efficiencies and minimising off-target errors.

## Opportunities and limitations of stem cell-derived embryo models

In part to overcome some of these limitations related to the accessibility and tractability of embryonic material, *in vitro* stem cell-based models have been developed that aim to replicate all or parts of an embryo (Fu et al., 2021; Rossant and Tam, 2021). Examples include non-integrated stem cell-based systems that model specific developmental tissues (including amnion and primordial germ cell formation; Shao et al., 2017), those that model specific stages (such as gastruloids; Moris et al., 2020), and those that model particular tissues or selections of tissues [such as segmentoids, somitoid and axioloids (Miao et al., 2023; Yamanaka et al., 2023), neural tube-like structures (Karzbrun et al., 2021), and organoids (Corsini and Knoblich, 2022)]. Other models, termed integrated models, aim to replicate the development of the entire conceptus, including the embryo and its extra-embryonic tissues, such as blastoids and ETX-based models (embryonic-trophoblast-extra-embryonic endoderm) (Amadei et al., 2022; Heidari Khoei et al., 2023; Kagawa et al., 2022; Lau et al., 2022; Liu et al., 2021; Tarazi et al., 2022; Yanagida et al., 2021; Yu et al., 2021).

The availability of integrated and non-integrated embryo models provides new opportunities for well-controlled interventional experiments and may provide insights into the biology of human embryos at this hiatus (Rossant and Tam, 2022). These models are well suited to investigate the ability of cells to undergo specific developmental processes, such as morphogenesis, self-organisation and patterning, without the complexity of the whole conceptus. Furthermore, their stem cell origins and potential for scalability also create new possibilities for chemical and genetic screens, physical manipulation and disease modelling, and are highly tractable for live-cell and genetic analyses.

However, many of these models are still in their infancy, and although the range and scope of available systems is wide and ever increasing, there are significant challenges that limit the questions they can address and the relevance of knowledge gained from them (Rossant and Tam, 2021). As a consequence, most models that are based on a reductionist approach are currently less suited to examining interactions between multiple tissue or lineage types. Future endeavours will need to meet these challenges to maximise their potential for yielding valuable insights into early development. Although not exhaustive, we discuss some of these technical challenges (Fig. 1) and potential solutions below.

### Technical limitations of stem cell-based embryo models

A common confounding factor of stem cell-based embryo models is the formation or inclusion of off-target cell types, i.e. cells that are not present in an embryo at the developmental stage that is being modelled. This was a problem that afflicted early blastoid studies, but now seems to be largely resolved (Kagawa et al., 2022; Zhao et al., 2021b preprint). However, an ongoing technical challenge involves the variability in hypoblast induction that is frequently observed when using current blastoid methods, where the localisation of hypoblast cells is usually accurate, but for reasons not fully understood, the number of hypoblast cells in blastoids varies beyond the range typically detected in blastocysts. Our limited understanding of how the hypoblast forms *in vivo* restricts our ability to optimise blastoid methodologies, although recent studies have employed innovations such as a two-step lineage induction system (Yu et al., 2022 preprint) and altering signalling modulators and engineering parameters (Vrij et al., 2022). Such examples confirm the need to define sample similarities not only by transcriptomic methods – including out-group samples to prevent over-integration (Sozen et al., 2022; Zhao et al., 2021b preprint) – but also based on additional characterisation, such as spatial localisation, subsequent cellular and tissue morphogenesis or, ideally, functional validation. In examples such as this, multi-lineage reporter cells could be useful for screening for factors that modulate the induction of different lineage fates in embryo-like models.

An area for further improvement is that of additional and better-characterised starting cell types for downstream differentiation and for assembling into embryo models. For example, efforts could focus on deriving human hypoblast stem cells directly from embryos, which has so far not been achieved, but their availability could lead to improvement of integrated models. This principle also extends more broadly to other cell types, such as trophoblast, amnion and mesenchymal cells, to better enable the formation of ‘assembloids’ involving multiple cell types (Lau et al., 2022). Concerted efforts to provide well-characterised and highly controlled culture conditions will also be important for enabling efficient and reproducible self-organising model generation, where small deviations can result in significant downstream effects.

Perhaps the most crucial challenge facing embryo-like models is that the developmental competence of integrated models is unknown, and there is no applicable technique to assess this. Attempts at the prolonged culture of human blastoids have so far been few, with studies demonstrating that blastoids fail to develop for more than a few days when attached to tissue culture plastic or to monolayers of endometrial cells (Kagawa et al., 2022; Yanagida et al., 2021; Yu et al., 2022 preprint). Clearly, improved culture systems that better mimic the implantation niche are required for maximising the research opportunities opened up by integrated models.

In parallel, studies in other species could shed light on the question of developmental competence. Mouse blastoids transferred into the receptive uteri of mice did not survive for more than 1 or 2 days (Rivron et al., 2018). Bovine blastoids transferred into bovine uteri triggered a pregnancy-associated signalling reaction in the recipient animals; however, no assessment of implantation or blastoid growth was reported (Pinzón-Arteaga et al., 2023). With further developments in the field, future efforts are likely to focus on the non-human primate; one recent example using cynomolgus monkeys has reportedly achieved implantation and pre-gastrulation development of blastoids after transfer into maternal uteri (Li et al., 2023). These are exciting advances, but given that we still only have competent naïve-state pluripotent stem cells from a limited number of species, and methods to generate and grow blastoids might vary to some extent between species, there are still substantial hurdles to be overcome.

### Robustness and reproducibility of stem cell-based embryo models

One challenge with any stem cell-based embryo model is to understand to what extent they can mimic key features of embryogenesis, and which features are not replicated *in vitro*. Additional data from human embryos themselves will be crucial in enabling accurate interpretation of observations from embryo models and enable researchers to assess both the quality and relevance of their models.

Benefits of stem cell-based embryo models include their tractability and scale, and the improved standardisation that these models should provide. An imperative is providing quantification when reporting results, as this information is crucial to understanding the ‘standard’ for the model. Furthermore, the models must be reproducible both within and between experiments, to gain statistical power to determine the differences of outcome after manipulation. In addition, many stem cell-based systems are subject to cell line differences, media composition dependence and unknown sources of variability, which reduces the overall robustness of the protocol. This problem is exacerbated by reliance on commercial products that may have batch-to-batch variation, are often proprietary in formulation and can lead to obstacles outside the control of researchers, such as product discontinuation or global supply-chain issues. Particularly for the putatively self-organising systems, where any deviation from an ideal set-point can strongly affect the experimental outcome, this dependence can create severe constraints to overcome.

Potential solutions to the reproducibility challenges abound, and it may help to look to fields outside biology and across disciplines. For example, studies have used bioengineering approaches, such as constraining 2D culture to balance the proportions of cell types (Warmflash et al., 2014), or microfluidic systems to control access to morphogens (Zheng et al., 2020). Likewise, scaffolds that support or shape large structures and assembloids that bring several systems together can devise more-complex and more-complete representations of embryonic processes (Gupta et al., 2021; Liu and Warmflash, 2021; Vianello and Lutolf, 2019). In addition, introducing vascularisation might be able to support the continued growth and development of structures for longer time periods, and the addition of supporting cells, such as immune cells, could improve tissue homeostasis (Zhao et al., 2021a). Similarly, biochemistry approaches to produce defined and standardised medium components, and computational advances, including machine learning to optimise protocols (Anand et al., 2023), have the potential to enhance our technological capabilities while improving reproducibility. It is vital that the field promotes and encourages openness and sharing to enable some of these technical challenges to be overcome quickly and equitably, and to help refine the model systems used across research.

## Perspectives

Besides the ethical and regulatory hurdles to studying early human development, there are technical constraints to consider. Some of these are remarkably challenging and will require a concerted effort to bring about real change. In our opinion, a key priority is to fully benchmark embryo-like models to embryos – this is not easy and has still not been carried out in many cases. Ideally, we need to established not only whether models are similar in their transcriptional profiles, but also whether the molecular pathways, regulatory mechanisms and tempo at which the models develop are conserved and can replicate the regulatory logic and dynamics that occur in the natural embryo. Determining whether or not they do, or whether they follow novel or atypical pathways through development, will still be of value, but knowing this status will inform the interpretation of the knowledge gained from these systems. Likewise, we need to analyse sufficient embryonic samples to establish the variability in natural embryos in order to assess the variability observed across the models. Presently, the burning questions are: what is the range of ‘normal’ values and parameters, and how can we build systems that are robust and reproducible, yet allow the cells to display this variability?

To fulfil this goal, we will need solutions to fill the gap in knowledge at 2-4 weeks of embryo development. One route forward might lie in being able to culture embryos beyond day 14, which would shed light on a developmental stage that is all but inaccessible. Before doing so, scientists will need to tackle the challenges of keeping high-quality embryonic material embedded in implantation-like conditions and capable of undergoing sustained development. Scientists must also engage meaningfully with regulatory bodies and with society for consideration of the scientific merit of the investigation, and the legal and ethical issues of human embryo research (Piotrowska, 2020; Fabbri et al., 2023). Such groundwork will be necessary for communicating the scientific merit of this research to policymakers and the public, in terms of what new knowledge this would bring and the potential healthcare benefits, which are important for subsequent regulatory and ethical decision making.

Alongside this, attention should be paid to establishing resources and frameworks that enable new opportunities for research and collaboration, both within science and in engaging more widely with stakeholders. This includes sustainable funding to material banking and resource collections, setting up interdisciplinary teams and networks, and new training opportunities. Efforts such as these would underpin research and lower barriers to teams entering this field. Such mechanisms facilitate growth of the research discipline by bringing in new ideas and widening engagement with human developmental biology.

## References

[DEV201797C1] Alanis-Lobato, G., Zohren, J., McCarthy, A., Fogarty, N. M. E., Kubikova, N., Hardman, E., Greco, M., Wells, D., Turner, J. M. A. and Niakan, K. K. (2021). Frequent loss of heterozygosity in CRISPR-Cas9-edited early human embryos. *Proc. Natl. Acad. Sci. U. S. A* 118, e2004832117. 10.1073/pnas.2004832117 34050011 PMC8179174

[DEV201797C2] Amadei, G., Handford, C. E., Qiu, C., De Jonghe, J., Greenfeld, H., Tran, M., Martin, B. K., Chen, D.-Y., Aguilera-Castrejon, A., Hanna, J. H. et al. (2022). Embryo model completes gastrulation to neurulation and organogenesis. *Nature* 610, 143-153. 10.1038/s41586-022-05246-3 36007540 PMC9534772

[DEV201797C3] Anand, G. M., Megale, H. C., Murphy, S. H., Weis, T., Lin, Z., He, Y., Wang, X., Liu, J. and Ramanathan, S. (2023). Controlling organoid symmetry breaking uncovers an excitable system underlying human axial elongation. *Cell* 186, 497-512. 10.1016/j.cell.2022.12.043 36657443 PMC10122509

[DEV201797C4] Arutyunyan, A., Roberts, K., Troulé, K., Wong, F. C. K., Sheridan, M. A., Kats, I., Garcia-Alonso, L., Velten, B., Hoo, R., Ruiz-Morales, E. R. et al. (2023). Spatial multiomics map of trophoblast development in early pregnancy. *Nature* 616, 143-151. 10.1038/s41586-023-05869-0 36991123 PMC10076224

[DEV201797C5] Bergmann, S., Penfold, C. A., Slatery, E., Siriwardena, D., Drummer, C., Clark, S., Strawbridge, S. E., Kishimoto, K., Vickers, A., Tewary, M. et al. (2022). Spatial profiling of early primate gastrulation in utero. *Nature* 609, 136-143. 10.1038/s41586-022-04953-1 35709828 PMC7614364

[DEV201797C6] Boroviak, T., Stirparo, G. G., Dietmann, S., Hernando-Herraez, I., Mohammed, H., Reik, W., Smith, A., Sasaki, E., Nichols, J. and Bertone, P. (2018). Single cell transcriptome analysis of human, marmoset and mouse embryos reveals common and divergent features of preimplantation development. *Development* 145, dev167833. 10.1242/dev.167833 30413530 PMC6240320

[DEV201797C7] Corsini, N. S. and Knoblich, J. A. (2022). Human organoids: New strategies and methods for analyzing human development and disease. *Cell* 185, 2756-2769. 10.1016/j.cell.2022.06.051 35868278

[DEV201797C8] Cui, G., Feng, S., Yan, Y., Wang, L., He, X., Li, X., Duan, Y., Chen, J., Tang, K., Zheng, P. et al. (2022). Spatial molecular anatomy of germ layers in the gastrulating cynomolgus monkey embryo. *Cell Rep.* 40, 111285. 10.1016/j.celrep.2022.111285 36044859

[DEV201797C9] Deglincerti, A., Croft, G. F., Pietila, L. N., Zernicka-Goetz, M., Siggia, E. D. and Brivanlou, A. H. (2016). Self-organization of the in vitro attached human embryo. *Nature* 533, 251-254. 10.1038/nature17948 27144363

[DEV201797C10] Dennis, S. J., Thomas, M. A., Williams, D. B. and Robins, J. C. (2006). Embryo morphology score on day 3 is predictive of implantation and live birth rates. *J. Assist. Reprod. Genet.* 23, 171-175. 10.1007/s10815-006-9027-3 16758347 PMC3454961

[DEV201797C11] Fabbri, M., Ginoza, M., Assen, L., Jongsma, K. and Isasi, R. (2023). Modeling policy development: examining national governance of stem-cell based embryo models. *Regen. Med.* 18, 155-168. 10.2217/rme-2022-0136 36601984

[DEV201797C12] Fogarty, N. M. E., McCarthy, A., Snijders, K. E., Powell, B. E., Kubikova, N., Blakeley, P., Lea, R., Elder, K., Wamaitha, S. E., Kim, D. et al. (2017). Genome editing reveals a role for OCT4 in human embryogenesis. *Nature* 550, 67-73. 10.1038/nature24033 28953884 PMC5815497

[DEV201797C13] Fu, J., Warmflash, A. and Lutolf, M. P. (2021). Stem-cell-based embryo models for fundamental research and translation. *Nat. Mater.* 20, 132-144. 10.1038/s41563-020-00829-9 33199861 PMC7855549

[DEV201797C14] Gupta, A., Lutolf, M. P., Hughes, A. J. and Sonnen, K. F. (2021). Bioengineering in vitro models of embryonic development. *Stem Cell Reports* 16, 1104-1116. 10.1016/j.stemcr.2021.04.005 33979597 PMC8185467

[DEV201797C59] Heidari Khoei, H., Javali, A., Kagawa, H., Sommer, T. M., Sestini, G., David, L., Slovakova, J., Novatchkova, M., Scholte Op Reimer, Y. and Rivron, N. (2023). Generating human blastoids modeling blastocyst-stage embryos and implantation. *Nat. Protoc.* 18, 1584-1620. 10.1038/s41596-023-00802-1 36792779 PMC7617227

[DEV201797C15] Ismaili M'hamdi, H., Rivron, N. C. and Asscher, E. C. (2022). Going high and low: on pluralism and neutrality in human embryology policy-making. *J. Med. Ethics*. 10.1136/jme-2022-108515 36600611

[DEV201797C16] Kagawa, H., Javali, A., Khoei, H. H., Sommer, T. M., Sestini, G., Novatchkova, M., Scholte Op Reimer, Y., Castel, G., Bruneau, A., Maenhoudt, N. et al. (2022). Human blastoids model blastocyst development and implantation. *Nature* 601, 600-605. 10.1038/s41586-021-04267-8 34856602 PMC8791832

[DEV201797C17] Karzbrun, E., Khankhel, A. H., Megale, H. C., Glasauer, S. M. K., Wyle, Y., Britton, G., Warmflash, A., Kosik, K. S., Siggia, E. D., Shraiman, B. I. et al. (2021). Human neural tube morphogenesis in vitro by geometric constraints. *Nature* 599, 268-272. 10.1038/s41586-021-04026-9 34707290 PMC8828633

[DEV201797C18] Lau, K. Y. C., Rubinstein, H., Gantner, C. W., Hadas, R., Amadei, G., Stelzer, Y. and Zernicka-Goetz, M. (2022). Mouse embryo model derived exclusively from embryonic stem cells undergoes neurulation and heart development. *Cell Stem Cell* 29, 1445-1458.e8. 10.1016/j.stem.2022.08.013 36084657 PMC9648694

[DEV201797C19] Li, J., Zhu, Q., Cao, J., Liu, Y., Lu, Y., Sun, Y., Li, Q., Huang, Y., Shang, S., Bian, X. et al. (2023). Cynomolgus monkey embryo model captures gastrulation and early pregnancy. *Cell Stem Cell* 30, 362-377.e7. 10.1016/j.stem.2023.03.009 37028403

[DEV201797C20] Liu, L. and Warmflash, A. (2021). Self-organized signaling in stem cell models of embryos. *Stem Cell Reports* 16, 1065-1077. 10.1016/j.stemcr.2021.03.020 33979594 PMC8185436

[DEV201797C21] Liu, X., Tan, J. P., Schröder, J., Aberkane, A., Ouyang, J. F., Mohenska, M., Lim, S. M., Sun, Y. B. Y., Chen, J., Sun, G. et al. (2021). Modelling human blastocysts by reprogramming fibroblasts into iBlastoids. *Nature* 591, 627-632. 10.1038/s41586-021-03372-y 33731926

[DEV201797C22] Lovell-Badge, R., Anthony, E., Barker, R. A., Bubela, T., Brivanlou, A. H., Carpenter, M., Charo, R. A., Clark, A., Clayton, E., Cong, Y. et al. (2021). ISSCR guidelines for stem cell research and clinical translation: the 2021 update. *Stem Cell Reports* 16, 1398-1408. 10.1016/j.stemcr.2021.05.012 34048692 PMC8190668

[DEV201797C23] Matthews, K. R. W. and Morali, D. (2020). National human embryo and embryoid research policies: a survey of 22 top research-intensive countries. *Regen. Med.* 15, 1905-1917. 10.2217/rme-2019-0138 32799737

[DEV201797C24] Matthews, K. R. W., Iltis, A. S., Marquez, N. G., Wagner, D. S., Robert, J. S., de Melo-Martín, I., Bigg, M., Franklin, S., Holm, S., Metzler, I. et al. (2021). Rethinking human embryo research policies. *Hastings Cent. Rep* 51, 47-51. 10.1002/hast.1215 PMC7986614 33630327

[DEV201797C25] Miao, Y., Djeffal, Y., De Simone, A., Zhu, K., Lee, J. G., Lu, Z., Silberfeld, A., Rao, J., Tarazona, O. A., Mongera, A. et al. (2023). Reconstruction and deconstruction of human somitogenesis in vitro. *Nature* 614, 500-508. 10.1038/s41586-022-05655-4 36543321 PMC10018515

[DEV201797C26] Moris, N., Anlas, K., van den Brink, S. C., Alemany, A., Schröder, J., Ghimire, S., Balayo, T., van Oudenaarden, A. and Martinez Arias, A. (2020). An in vitro model of early anteroposterior organization during human development. *Nature* 582, 410-415. 10.1038/s41586-020-2383-9 32528178

[DEV201797C27] Nakamura, T., Okamoto, I., Sasaki, K., Yabuta, Y., Iwatani, C., Tsuchiya, H., Seita, Y., Nakamura, S., Yamamoto, T. and Saitou, M. (2016). A developmental coordinate of pluripotency among mice, monkeys and humans. *Nature* 537, 57-62. 10.1038/nature19096 27556940

[DEV201797C28] Nakamura, T., Yabuta, Y., Okamoto, I., Sasaki, K., Iwatani, C., Tsuchiya, H. and Saitou, M. (2017). Single-cell transcriptome of early embryos and cultured embryonic stem cells of cynomolgus monkeys. *Sci Data* 4, 170067. 10.1038/sdata.2017.67 28649393 PMC5477564

[DEV201797C29] Pereira Daoud, A. M., Popovic, M., Dondorp, W. J., Trani Bustos, M., Bredenoord, A. L., Chuva de Sousa Lopes, S. M., van den Brink, S. C., Roelen, B. A. J., de Wert, G. M. W. R. and Heindryckx, B. (2020). Modelling human embryogenesis: embryo-like structures spark ethical and policy debate. *Hum. Reprod. Update* 26, 779-798. 10.1093/humupd/dmaa027 32712668

[DEV201797C30] Pinzón-Arteaga, C. A., Wang, Y., Wei, Y., Li, L., Ribeiro Orsi, A. E., Scatolin, G., Liu, L., Sakurai, M., Ye, J., Yu, L. et al. (2023). Bovine blastocyst like structures derived from stem cell cultures. *Cell Stem Cell* 30, 611-616. 10.1016/j.stem.2023.04.003 37146582 PMC10230549

[DEV201797C31] Piotrowska, M. (2020). Avoiding the potentiality trap: thinking about the moral status of synthetic embryos. *Monash Bioethics Rev* 38, 166-180. 10.1007/s40592-019-00099-5 31741321

[DEV201797C32] Rawlings, T. M., Makwana, K., Taylor, D. M., Molè, M. A., Fishwick, K. J., Tryfonos, M., Odendaal, J., Hawkes, A., Zernicka-Goetz, M., Hartshorne, G. M. et al. (2021). Modelling the impact of decidual senescence on embryo implantation in human endometrial assembloids. *Elife* 10, e69603. 10.7554/eLife.69603 34487490 PMC8523170

[DEV201797C33] Rivron, N. C., Frias-Aldeguer, J., Vrij, E. J., Boisset, J.-C., Korving, J., Vivié, J., Truckenmüller, R. K., van Oudenaarden, A., van Blitterswijk, C. A. and Geijsen, N. (2018). Blastocyst-like structures generated solely from stem cells. *Nature* 557, 106-111. 10.1038/s41586-018-0051-0 29720634

[DEV201797C34] Rossant, J. and Tam, P. P. L. (2021). Opportunities and challenges with stem cell-based embryo models. *Stem Cell Reports* 16, 1031-1038. 10.1016/j.stemcr.2021.02.002 33667412 PMC8185371

[DEV201797C35] Rossant, J. and Tam, P. P. L. (2022). Early human embryonic development: Blastocyst formation to gastrulation. *Dev. Cell* 57, 152-165. 10.1016/j.devcel.2021.12.022 35077679

[DEV201797C36] Samorinha, C., Pereira, M., Machado, H., Figueiredo, B. and Silva, S. (2014). Factors associated with the donation and non-donation of embryos for research: a systematic review. *Hum. Reprod. Update* 20, 641-655. 10.1093/humupd/dmu026 24907125

[DEV201797C37] Shahbazi, M. N., Jedrusik, A., Vuoristo, S., Recher, G., Hupalowska, A., Bolton, V., Fogarty, N. N. M., Campbell, A., Devito, L., Ilic, D. et al. (2016). Self-organization of the human embryo in the absence of maternal tissues. *Nat. Cell Biol.* 18, 700-708. 10.1038/ncb3347 27144686 PMC5049689

[DEV201797C38] Shao, Y., Taniguchi, K., Townshend, R. F., Miki, T., Gumucio, D. L. and Fu, J. (2017). A pluripotent stem cell-based model for post-implantation human amniotic sac development. *Nat. Commun.* 8, 208. 10.1038/s41467-017-00236-w 28785084 PMC5547056

[DEV201797C39] Siriwardena, D. and Boroviak, T. E. (2022). Evolutionary divergence of embryo implantation in primates. *Philos. Trans. R. Soc. Lond. B Biol. Sci.* 377, 20210256. 10.1098/rstb.2021.0256 36252209 PMC9574630

[DEV201797C40] Sozen, B., Conkar, D. and Veenvliet, J. V. (2022). Carnegie in 4D? Stem-cell-based models of human embryo development. *Semin. Cell Dev. Biol.* 131, 44-57. 10.1016/j.semcdb.2022.05.023 35701286

[DEV201797C41] Stamatiadis, P., Boel, A., Cosemans, G., Popovic, M., Bekaert, B., Guggilla, R., Tang, M., De Sutter, P., Van Nieuwerburgh, F., Menten, B. et al. (2021). Comparative analysis of mouse and human preimplantation development following POU5F1 CRISPR/Cas9 targeting reveals interspecies differences. *Hum. Reprod* 36, 1242-1252. 10.1093/humrep/deab027 33609360

[DEV201797C61] Stamatiadis, P., Cosemans, G., Boel, A., Menten, B., De Sutter, P., Stoop, D., Chuva de Sousa Lopes, S.M., Lluis, F., Coucke, P. and Heindryckx, B. (2022). TEAD4 regulates trophectoderm differentiation upstream of CDX2 in a GATA3-independent manner in the human preimplantation embryo. Hum. Reprod. 37, 1760-1773. 10.1093/humrep/deac138 35700449

[DEV201797C42] Tarazi, S., Aguilera-Castrejon, A., Joubran, C., Ghanem, N., Ashouokhi, S., Roncato, F., Wildschutz, E., Haddad, M., Oldak, B., Gomez-Cesar, E. et al. (2022). Post-gastrulation synthetic embryos generated ex utero from mouse naive ESCs. *Cell* 185, 3290-3306.e25. 10.1016/j.cell.2022.07.028 35988542 PMC9439721

[DEV201797C43] Tyser, R. C. V., Mahammadov, E., Nakanoh, S., Vallier, L., Scialdone, A. and Srinivas, S. (2021). Single-cell transcriptomic characterization of a gastrulating human embryo. *Nature* 600, 285-289. 10.1038/s41586-021-04158-y 34789876 PMC7615353

[DEV201797C44] Vianello, S. and Lutolf, M. P. (2019). Understanding the mechanobiology of early mammalian development through bioengineering models. *Dev. Cell* 48, 751-763. 10.1016/j.devcel.2019.02.024 30913407

[DEV201797C45] Vrij, E. J., Reimer, S. O., Roa Fuentes, Y. S., Misteli Guerreiro, L., Holzmann, I., Frias Aldeguer, V., Sestini, J., Koo, G., Kind, B.-K., van Blitterswijk, J. (2022). A pendulum of induction between the epiblast and extra-embryonic endoderm supports post-implantation progression. *Development* 149, dev192310. 10.1242/dev.192310 35993866 PMC9534490

[DEV201797C46] Warmflash, A., Sorre, B., Etoc, F., Siggia, E. D. and Brivanlou, A. H. (2014). A method to recapitulate early embryonic spatial patterning in human embryonic stem cells. *Nat. Methods* 11, 847. 10.1038/nmeth.3016 24973948 PMC4341966

[DEV201797C47] Xiang, L., Yin, Y., Zheng, Y., Ma, Y., Li, Y., Zhao, Z., Guo, J., Ai, Z., Niu, Y., Duan, K. et al. (2020). A developmental landscape of 3D-cultured human pre-gastrulation embryos. *Nature* 577, 537-542. 10.1038/s41586-019-1875-y 31830756

[DEV201797C48] Xu, Y., Zhang, T., Zhou, Q., Hu, M., Qi, Y., Xue, Y., Nie, Y., Wang, L., Bao, Z. and Shi, W. (2023). A single-cell transcriptome atlas profiles early organogenesis in human embryos. *Nat. Cell Biol* 25, 604-615. 10.1038/s41556-023-01108-w 36928764

[DEV201797C49] Yamanaka, Y., Hamidi, S., Yoshioka-Kobayashi, K., Munira, S., Sunadome, K., Zhang, Y., Kurokawa, Y., Ericsson, R., Mieda, A., Thompson, J. L. et al. (2023). Reconstituting human somitogenesis in vitro. *Nature* 614, 509-520. 10.1038/s41586-022-05649-2 36543322

[DEV201797C50] Yanagida, A., Spindlow, D., Nichols, J., Dattani, A., Smith, A. and Guo, G. (2021). Naive stem cell blastocyst model captures human embryo lineage segregation. *Cell Stem Cell* 28, 1016-1022.e4. 10.1016/j.stem.2021.04.031 33957081 PMC8189436

[DEV201797C51] Yu, L., Wei, Y., Duan, J., Schmitz, D. A., Sakurai, M., Wang, L., Wang, K., Zhao, S., Hon, G. C. and Wu, J. (2021). Blastocyst-like structures generated from human pluripotent stem cells. *Nature* 591, 620-626. 10.1038/s41586-021-03356-y 33731924

[DEV201797C52] Yu, L., Ezashi, T., Wei, Y., Duan, J., Logsdon, D., Zhan, L., Nahar, A., Arteaga, C. P., Liu, L., Stobbe, C. et al. (2022). Large scale production of human blastoids amenable to modeling blastocyst development and maternal-fetal crosstalk. *bioRxiv* 10.1101/2022.09.14.507946 37683605

[DEV201797C53] Zhai, J., Guo, J., Wan, H., Qi, L., Liu, L., Xiao, Z., Yan, L., Schmitz, D. A., Xu, Y., Yu, D. et al. (2022). Primate gastrulation and early organogenesis at single-cell resolution. *Nature* 612, 732-738. 10.1038/s41586-022-05526-y 36517595 PMC9771819

[DEV201797C54] Zhang, D., Deng, Y., Kukanja, P., Agirre, E., Bartosovic, M., Dong, M., Ma, G., Ma, S., Bao, S., Liu, Y. et al. (2023). Spatial epigenome-transcriptome co-profiling of mammalian tissues. *Nature* 616, 113-122. 10.1038/s41586-023-05795-1 36922587 PMC10076218

[DEV201797C55] Zhao, X., Xu, Z., Xiao, L., Shi, T., Xiao, H., Wang, Y., Li, Y., Xue, F. and Zeng, W. (2021a). Review on the vascularization of organoids and organoids-on-a-hip. *Front Bioeng Biotechnol* 9, 637048. 10.3389/fbioe.2021.637048 33912545 PMC8072266

[DEV201797C56] Zhao, C., Reyes, A. P., Schell, J. P., Weltner, J., Ortega, N. M., Zheng, Y., Björklund, Å. K., Rossant, J., Fu, J., Petropoulos, S. et al. (2021b). Reprogrammed blastoids contain amnion-like cells but not trophectoderm. *bioRxiv* 10.1101/2021.05.07.442980

[DEV201797C57] Zheng, Y., Shao, Y. and Fu, J. (2020). A microfluidics-based stem cell model of early post-implantation human development. *Nat. Protoc* 16, 309-326. 10.1038/s41596-020-00417-w 33311712

[DEV201797C58] Zuccaro, M. V., Xu, J., Mitchell, C., Marin, D., Zimmerman, R., Rana, B., Weinstein, E., King, R. T., Palmerola, K. L., Smith, M. E. et al. (2020). Allele-specific chromosome removal after Cas9 cleavage in human embryos. *Cell* 183, 1650-1664. 10.1016/j.cell.2020.10.025 33125898

